# Complete genome sequence of Azrael100, a V cluster mycobacteriophage

**DOI:** 10.1128/MRA.00684-23

**Published:** 2023-09-21

**Authors:** Edith Erika Machowski, Christopher Shawn Ealand, Olivia Jacobs, Bavesh Davandra Kana

**Affiliations:** 1 DSI/NRF Centre of Excellence for Biomedical TB Research, Faculty of Health Sciences, University of the Witwatersrand, National Health Laboratory Service, Johannesburg, South Africa; Queens College, Queens, New York, USA

**Keywords:** mycobacteriophage, *Mycobacterium smegmatis*

## Abstract

Azrael100, a cluster V siphoviral mycobacteriophage, was isolated from a garden in Johannesburg, South Africa. It can infect and lyse *Mycobacterium smegmatis* mc^2^155. The double-stranded DNA genome contains 78,063 base pairs with a GC content of 56.9%, with 141 predicted open reading frames, 23 tRNAs, and one tmRNA.

## ANNOUNCEMENT

Mycobacteriophage (MP) therapy has shown promise in augmenting current regimens against mycobacterial diseases (1–4). Bacteria can become resistant to bacteriophages, and thus a diverse pool of available phages is required to ensure flexibility to generate tailored cocktails (5).

**Fig 1:**
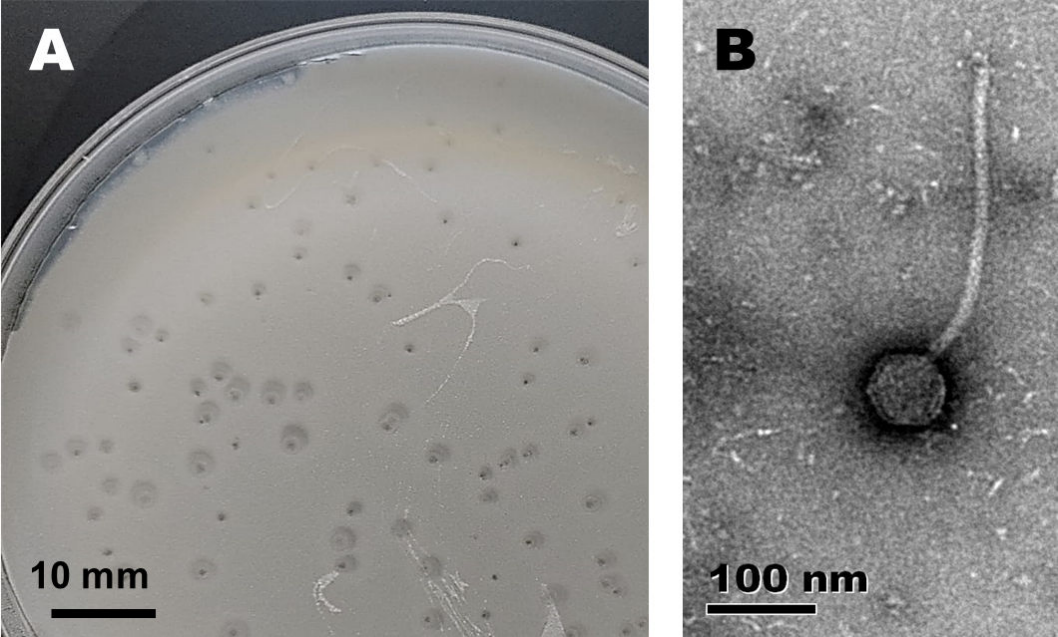
Morphological characterization of mycobacteriophage Azrael100. (**A**) Plaques produced on a lawn of *M. smegmatis* mc^2^155 grown on 7H10 medium on a petri dish (90 mm diameter). They are clear, ca. 1 to 3 mm in diameter with a cornet-shaped halo. (**B**) Transmission electron micrograph of virion morphology (stained with 1% uranyl acetate). Azrael100 contains a ~75-nm-wide head and a non-contractile tail with a length of ~294 nm.

We isolated Azrael100 from compost soil in Johannesburg, South Africa (June 2021; GPS coordinates—26.188773°, 28.005199°). Soil was resuspended in 10 mL of mycobacteriophage buffer and vortexed for 2 min. After settling, the liquid was filter-sterilized (0.22 µm) and used to infect *Mycobacterium smegmatis* mc^2^155 to obtain plaques. Briefly, 450 µL of bacterial cells were washed twice in MP buffer, and 50 µL of filtered lysate was added for phage adsorption. The mixture was poured as an overlay and incubated for 48 h at 37°C (6). A clear, small plaque (ca. 1–3 mm, Fig. 1A) was picked for purification and propagation. High titer phage lysate was used for negative staining transmission electron microscopy and genomic DNA extraction (Wizard Genomic DNA Purification Kit, Promega).

Library preparation was performed using the NEBNext Ultra II FS Kit (New England Biolabs). DNA was indexed and sequenced on the Illumina NextSeq500 platform, using a NextSeq (300 cycle) Kit as previously described (7). A total of 601,246 reads (2 × 150 bp paired-end reads) were generated and trimmed (Illumina Experiment Manager v1.9 with default settings) before genome assembly. A single mycobacteriophage contig was assembled and assessed for quality, completeness, accuracy, and genomic termini, using Newbler (V2.9) and Consed (V29.0) with default parameters. The approximate coverage level was 2,198-fold. Whole-genome nucleotide BLASTn alignments were performed at https://blast.ncbi.nlm.nih.gov/ and https://phagesdb.org/blast/. The genome was annotated using DNA Master (v5.23.6; http://phagesdb.org/DNAMaster/) and refined with the following databases and software with default parameters unless otherwise stated: GeneMark (v2.5p) (8), Glimmer (v3.07) (9), Phamerator (https://phamerator.org) (10), and Aragorn (11). The tRNAscan-SE (v2.0) (12, 13) software tool was modified as follows: Sequence source: Bacterial; Search mode: Infernal without HMM; Extended Options: Check “Disable pseudo gene checking”; Check “Show primary and secondary structure components to scores”; and Genetic Code for tRNA isotype Prediction: Universal and a Score cut-off of 17.

The morphotype of Azrael100 is siphoviral, with the icosahedral head diameter and tail fiber length measuring 75 nm and 294 nm, respectively (Fig. 1B). The double stranded DNA genome of 78,063 base pairs is circularly permuted, with a 3’ sticky overhang of 11 bp (ACCACTGCAAC) and a GC content of 56.9%. It has >98.96% similarity with the other four cluster V mycobacteriophages, i.e., Cosmo (GenBank accession number: KP027195), MaryV (MN585992), EniyanLRS (KY385381), and Wildcat (NC_008206). There are 141 predicted open reading frames (ORFs) of which 99 (61%) are hypothetical proteins. There are 23 operonic tRNAs and one tmRNA, a possible indication that Azrael100 might infect a broad range of host strains (14). ORFs with homology to other known genes encode, among others, structural elements (e.g., capsid proteins and assembly protease, head-to-tail adaptors, neck protein, tail terminator, major tail, and tape measure proteins) and DNA modifying elements (e.g., PolyA polymerase, terminase, DnaB-like helicase, WhiB family transcription factor, and DNA polymerase).

## Data Availability

The Azrael100 genome sequence is available at GenBank under accession number OR199846 and the raw sequence reads under SRA accession number SRR24526040.
